# Assessing a Community Health Worker-Facilitated, Digitally Delivered, Family-Centered Diabetes Management Program: Single-Arm Quasi-Experimental Study

**DOI:** 10.2196/79032

**Published:** 2025-10-06

**Authors:** Zenong Yin, Vanessa L Errisuriz, Heather Cuevas, Bertha E Flores, Laura Delfausse, Christina Galvan, Jing Wang, Chengdong Li, Renata Morfin, Shiyu Li, Maysa Sapargeldiyeva, Giliane Yza Muyna, Minyu Zhang, Vanessa Sweet, Deborah Parra-Medina

**Affiliations:** 1 College of Health, Community and Policy University of Texas at San Antonio San Antonio, TX United States; 2 Department of Public Health Santa Clara University Santa Clara, CA United States; 3 School of Nursing University of Texas at Austin Austin, TX United States; 4 School of Nursing University of Texas Health Science Center San Antonio San Antonio, TX United States; 5 Latino Research Institute University of Texas at Austin Austin, TX United States; 6 College of Nursing Florida State University Tallahassee, FL United States; 7 School of Kinesiology Louisiana State University Baton Rouge, LA United States; 8 Greater Austin YMCA Austin, TX United States; 9 Department of Family Medicine University of Colorado Denver Anschutz Medical Campus Aurora, CO United States

**Keywords:** health equity, academic-community partnership, community health workers, diabetes self-management, diabetes education, diabetes support

## Abstract

**Background:**

The high prevalence of type 2 diabetes (T2D) and associated complications disproportionately affect low-income Latino populations, who also experience disparities in diabetes self-management (DSM), including poor medication adherence, physical activity, diet, and glycemic control.

**Objective:**

This study examined, through an academic-community partnership, the effectiveness of ¡Salud, Salud! (an evidence-based, family-centered diabetes self-management education and support [DSMES] program) on primary (glycemic control and quality of life) and secondary (social, psychological, and behavioral factors related to T2D management) outcomes among low-income Latino adults with T2D or prediabetes.

**Methods:**

In total, 81 adults (mean age 48.90 years, SD 12.57; n=57, 70.4%, female; n=66, 81.5%, Latino) with T2D or prediabetes were enrolled in a 12-week, single-arm quasi-experimental study conducted in two Central Texas Young Men’s Christian Association (YMCA) locations. ¡Salud, Salud! incorporated individual coaching by community health workers (CHWs), online family-centered DSMES training lessons, and a YMCA family membership. The delivery of ¡Salud, Salud! was supported and facilitated by digital technologies, including a dashboard to deliver intervention content and monitor participants’ engagement in intervention activities. Outcomes measured at baseline and 12 weeks (ie, postintervention) included hemoglobin A1c (HbA1c); quality of life; anthropometrics; self-reported physical activity and diet; mindfulness; perceived stress; and diabetes-related knowledge, self-efficacy, and support. Participant engagement in program activities was assessed via four index variables that underlay multiple dimensions of influences on ¡Salud, Salud! uptake: family engagement and support, participation in self-management education, program support and facilitation, and participation in self-monitoring. Paired t-tests and McNemar chi-square tests were used to examine the change in outcomes from baseline to 12 weeks. The number of program activities participants completed for each engagement index variable was converted to percentages to estimate the mean proportion of activities completed.

**Results:**

In total, 48 (59.3%) participants completed the 12-week posttest. At the end of the program, participants demonstrated a marginally significant reduction in HbA1c (–0.30%, *P*≤.09) and a significant increase in participants reporting good-to-excellent health from baseline (n=19, 39.6%) to posttest (n=28, 58.3%; *P*≤.003). There were significant reductions in body weight (–1.30 kg, *P*=.02), body fat percentage (–1.26%, *P*=.01), perceived stress (–0.28, *P*=.02), added sugar intake (–2.15 teaspoons/day, *P*=.001), and time spent sedentary per week (–70.27 minutes, *P*=.003) from baseline to posttest. Mindfulness increased significantly (2.21, *P*=.01). Participant engagement in ¡Salud, Salud! varied, with participants exhibiting a high completion rate in program support and facilitation activities (88%) and a moderate-to-low completion rate in self-management training (66%), self-monitoring (56%), and family engagement and support (49%) activities.

**Conclusions:**

¡Salud, Salud! shows promising preliminary effects on key diabetes-related outcomes. Future research should investigate how to enhance participant engagement and optimize uptake of evidence-based T2D self-management practices among low-income Latino adults with diabetes.

## Introduction

The prevalence of type 2 diabetes (T2D) in the United States is 14.0% [1], while prediabetes affects 48% of US adults [2]. In 2017, the economic burden of diabetes reached US $327.2 billion, translating to an annual cost of US $13,240 per patient [3]. T2D accounts for 90%-95% of diabetes cases [4] and disproportionately affects low-income and racial/ethnic minority communities, particularly Hispanics/Latinos (referred to hereafter as Latinos)—the largest racial/ethnic minority group in the United States, representing 18% of the population. During 2013-2016, T2D prevalence was 19.8% in Latinos compared to 12.4% in non-Latino Whites [1], with recent data indicating that diabetes incidence has decreased in non-Latino Whites but not in other racial and ethnic groups [5].

Low-income Latino populations also experience significant disparities in diabetes self-management education and support (DSMES) outcomes [1,6]. These disparities manifest as lower diabetes medication adherence rates [7], reduced likelihood of meeting recommended physical activity and dietary guidelines [1], and poorer glycemic control. Notably, only 8.4% of Latinos in the Hispanic Community Health Study/Study of Latinos met the goals for glycemic control [8]. These challenges contribute to higher risks for diabetes complications and mortality compared to non-Latino Whites.

Multiple factors influence diabetes self-management (DSM) practices among low-income minority individuals with T2D and their families [9,10]. These factors can be categorized into three primary domains: personal and interpersonal, social determinants of health, and cultural and social barriers [11,12]. Key personal and interpersonal factors include DSM knowledge and skills, DSM efficacy, adherence to DSM behaviors, T2D-related stress and anger, and family and friend support. Additional factors include time conflicts with family responsibilities and use of digital technologies [13]. Access to DSMES is often impeded by social determinants, such as environmental and transportation barriers, lack of health insurance, language and culture incongruence with health care providers, concerns about documentation status, economic hardships, and limited health literacy. Cultural and social barriers also deter DSMES among low-income racial/ethnic minority populations, including constrained financial resources, acculturative stress, physically demanding occupations, family values, and culturally specific preferences for food and physical activity [14].

The persistent disparities in diabetes outcomes among Latinos, coupled with the unique cultural and social factors affecting the uptake of evidence-based DSM practices, highlight the need for innovative, culturally responsive approaches to diabetes management [15-17]. Family-centered interventions may be particularly relevant for Latino populations, given the cultural emphasis on *familismo*—the central role of family in daily life and health decisions [18]. Additionally, the integration of community health workers (CHWs) and digital technologies offers promising strategies to overcome social determinant–related barriers to care, while maintaining cultural concordance [13,19].

To address these needs, we developed and evaluated *¡Salud, Salud!*, an evidence-based, family-centered DSMES program that leverages both CHW facilitation and digital technologies. This program aimed to improve glycemic control and health-related quality of life (HRQoL) for people with T2D and prediabetes from low-income predominantly Latino families in Austin, Texas. The study evaluated the program’s impact on primary outcomes (glycemic control and quality of life) and secondary outcomes (social, psychological, and behavioral factors related to DSM). We hypothesized that participants would demonstrate significant reductions in hemoglobin A1c (HbA1c) and improvements in self-reported general health following the 12-week intervention.

## Methods

### Study Design

A quasi-experimental, single-group, pretest-posttest design was used to evaluate the preliminary effectiveness of the ¡Salud, Salud! program in improving glycemic control and HRQoL. The study planned to recruit 98 dyads. Participant dyads consisted of one individual with T2D or prediabetes and a family member or fictive kin who lived with them or in the same geographic area. Participants who did not have a family member or fictive kin were permitted to enroll in the study. Findings related to the implementation of ¡Salud, Salud! have been published elsewhere [20].

### Study Setting

The geographic focus for this study was the city of Austin in Travis County, Texas. In Travis County, 33.9% of the population is Latino, 7.8% Black, and 7.6% Asian, and 31.1% are speakers of a non-English language (most commonly Spanish). Social disadvantage and vulnerability in Austin are concentrated in an area known as the Eastern Crescent, a geographic area of the city that has experienced historical economic disinvestment that has shaped access to critical resources needed to maintain health for community residents. The Eastern Crescent has a high proportion of Latino residents (53.8%), and almost one-third live below the poverty level, lack personal transportation, and have no health insurance. The prevalence of obesity (40%) and diabetes (16%) in the Eastern Crescent is higher than in the county and state. Given the high concentration of economically disadvantaged Latinos with limited English proficiency in the Eastern Crescent, we focused our efforts on providing the program to this subpopulation. In consultation with our community partner, the Young Men’s Christian Association (YMCA) of Austin, we identified two YMCA sites located within the Eastern Crescent to deliver the program.

### Recruitment and Eligibility

Multiple strategies were used to recruit individuals with T2D or prediabetes into the program. Flyers were distributed and posted at various community organizations that served residents of the Eastern Crescent (eg, safety net health clinics, food banks, churches). The program was also promoted through the YMCA’s e-newsletters, website, and social media platforms, such as Facebook. All flyers and promotional materials were available in English and Spanish and included a QR code linking to the YMCA’s ¡Salud, Salud! program webpage. This webpage included a link to the study’s online eligibility screening form hosted on REDCap, a secure web platform for data management. Interested individuals could either complete the eligibility screening form directly or provide their contact information (ie, name, telephone number, email address) if they preferred to speak with research staff. In such cases, research staff followed up over the phone to conduct eligibility screening. In addition, bilingual (English and Spanish) CHWs and research staff conducted in-person outreach by tabling at safety net clinics, community events, and YMCA sites. Using internet-enabled tablets, they informed attendees about the program and conducted onsite eligibility screenings in English or Spanish. All eligible individuals were referred to research staff to provide informed consent and complete baseline assessments.

Individuals were eligible if they reported that they had physician-diagnosed T2D or prediabetes, were at least 12 years old, had a smartphone, lived in Austin, and were willing to travel to program sites. Individuals were excluded from participation if they could not speak and read English or Spanish; were pregnant, nursing, or planning to become pregnant during the study; planned to relocate in the next 6 months; had a serious illness or unstable condition requiring supervision of diet and activity beyond T2D control; had a severe psychiatric illness that would prohibit following self-monitoring recommendations; or were unable or unwilling to complete survey instruments and assessment procedures. Participants were highly encouraged, but not required, to identify a family member, fictive kin, or community member to participate in the program with them as a support person. The support person needed to be at least 18 years old, have a smartphone, live in the same county as the participant, and be able and willing to complete survey instruments and assessment procedures. Enrollment was not limited by sex, sexual orientation, race, or ethnicity.

We aimed to recruit 98 participants with T2D or prediabetes. This sample size was calculated based on detecting a change in HbA1c at 12 weeks. Using data from a previous study showing a mean 0.1% HbA1c decrease (SD 0.29%) [21], we determined that 68 participants would provide 80% power with a 5% significance level, accounting for 30% attrition at the posttest.

### Intervention

**Figure 1:**
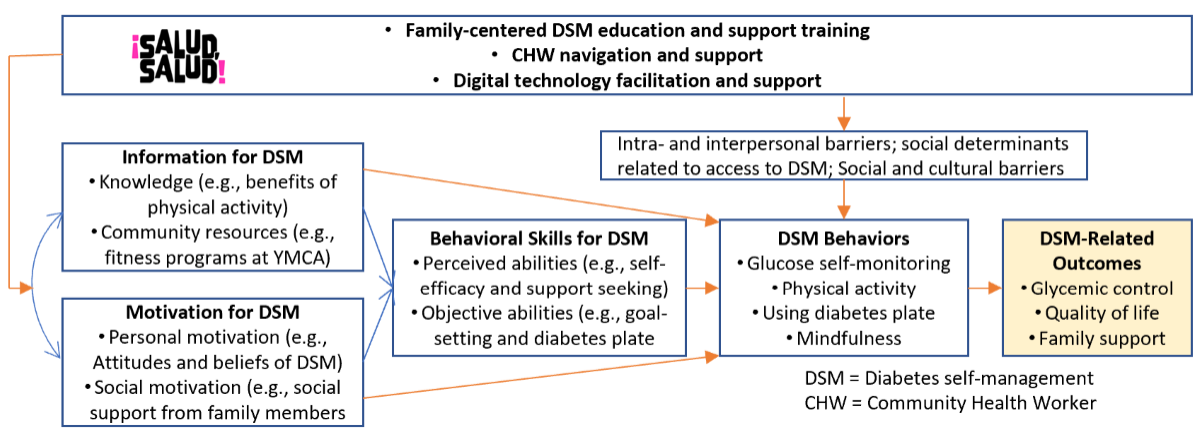
¡Salud, Salud! conceptual model.

To promote the uptake and application of DSMES practices by participants and their support persons, the information-motivation-behavioral skills (IMB) model was used to guide development of ¡Salud, Salud! (see Figure 1 for the conceptual model). According to the IMB model, successful diabetes management depends on three factors: Individuals with diabetes must be (1) informed of the knowledge and benefits of DSM, (2) motivated by personal (eg, beliefs and commitment) and social (eg, family support) factors related to DSM, and (3) equipped with behavioral skills to engage in DSM [3,4]. ¡Salud, Salud! also uses evidence-based strategies and practices from the “Standards of Medical Care in Diabetes” [22] and the DSMES Toolkit [19]. Evidence-based strategies included the use of mobile health (mHealth) and CHWs (nonmedical public health workers who connect communities to health care and social service providers) to deliver the program and engage participants with T2D and their designated support persons. Table 1 shows the components of the ¡Salud, Salud! intervention.

**Table 1:** ¡Salud, Salud! intervention components.

Component	Content and delivery
Family-centered DSMES^a^ training	Onsite, in-person program orientation for participants and their support persons with a CHW^b^ 1 in-person or virtual nutrition counseling session with a nutrition educator 8 online, interactive DSMES training lessons for participants and their support persons to increase family support and uptake of DSM^c^ practices
CHW navigation and support	12 weekly coaching sessions for support and problem solving with a CHW Goal setting and action planning for DSM practices with a CHW
Digital technology facilitation and support for DSM via the Connected Health Dashboard	Weekly online lesson delivery and lesson completion tracking Text message delivery (motivation, tips, and program reminders) Weekly self-monitoring survey delivery and tracking

^a^DSMES: diabetes self-management education and support.

^b^CHW: community health worker.

^c^DSM: diabetes self-management.

Participants and their designated support persons (when available) were introduced to the ¡Salud, Salud! program through a one-time, in-person orientation session held at a YMCA program site of their choosing. During this session, they met with their CHWs and learned about program goals and expectations. Participants were registered for a free 6-month YMCA family membership (ie, 2 adults and up to 4 children). The YMCA membership included access to all in-person and virtual YMCA programming, as well as a complementary personal training session. One week following the orientation, participants attended a one-on-one nutrition counseling session, which included an introduction to the American Diabetes Association’s Diabetes Plate Method and individualized dietary counseling. The CHWs also introduced participants to the digital tools they would use during the program, including the YMCA mobile app, the Connected Health Dashboard [23], and the Insight Timer app [24]. Insight Timer is a popular meditation app that offers a wide variety of guided meditations, music tracks, and talks from mindfulness experts in various languages, including English and Spanish, to help users reduce stress, improve sleep, and enhance overall well-being [24].

The delivery of self-guided DSMES was facilitated by an online mHealth platform, the Connected Health Dashboard [23]. The dashboard is designed to allow participants the flexibility to view DSMES online lessons and perform self-monitoring activities at their convenience. All participants received eight narrated and interactive core lessons. The core lessons have been designed to provide strategies that engage the whole family in health practices that could positively impact participants’ DSM and overall family health and well-being. A supplemental resource library of behavioral lessons was made available so that participants could receive additional training and support tailored to their individual behavioral change needs (eg, smoking, sleep, mindfulness). All content available on the dashboard could be viewed in English or Spanish, based on participant preference.

Each week, participants received a text message with a link to complete a brief self-monitoring survey to track the following behaviors: physical activity, healthy eating, mindfulness practice, and blood glucose monitoring. The dashboard linked to and displayed data collected from various digital sources (eg, lesson completion, surveys) so that participants and CHWs could monitor progress toward behavioral goals. The dashboard was also used to disseminate program text messages developed to provide informational and motivational support and enhance participant engagement with the dashboard.

CHWs provided personalized support to program participants via 11 weekly one-on-one encounters. These encounters were offered in person or remotely based on participants’ preferences. During these encounters, participants selected and set SMART goals for two behavioral focus areas from the following: physical activity, healthy eating, mindfulness practice, and blood glucose monitoring. CHWs used motivational interviewing techniques to guide participants toward achieving these goals. CHWs were highly trained, having completed the CHW certification program established by the Texas Department of State Health Services [5], the Diabetes Paraprofessional Level 2 certification program established by the Association of Diabetes Care & Education Specialists [6], and a 3-day training (~24 hours) offered by study research staff about how to deliver the ¡Salud, Salud! intervention. Training topics covered by study research staff included motivational interviewing techniques, review of interactive online DSMES modules, and use of the Connected Health Dashboard.

### Study Measurements

Assessment of study outcomes followed standardized data collection protocols conducted at baseline and immediately following completion of the 12-week program. Research assistants contacted participants over the phone and via text messages to schedule appointments to complete data collection in person at YMCA sites. Participants completed self-reported questionnaires related to quality of life, DSMES-related psychosocial factors and behaviors, and demographics. All questionnaires are available in English and Spanish, are reliable and valid, and have been used with Latino, Spanish-speaking, or economically disadvantaged populations. Standardized measurements of participant blood glucose levels and anthropometrics were also conducted in person by trained research assistants at YMCA sites. No incentives were provided for completing assessments. For the study, we included only data from participants with T2D or prediabetes.

#### Primary Outcomes

##### HbA1c Tests

Blood glucose levels were assessed using the A1CNow+, a certified method listed in the National Glycated Hemoglobin Standardization Program, that analyzes average blood glucose levels over a 3-month period [7]. Results are interpreted as follows: normal range (<5.7%), prediabetes (5.7%-6.4%), and T2D (≥6.5%).

##### Health-Related Quality of Life

Participants’ HRQoL was measured with the four core items of the CDC Healthy Days HRQOL Survey, which is designed to assess an individual’s perceived sense of well-being through a brief questionnaire [8]. Participants rated their general health on a scale from poor to excellent and reported the number of days over the past month during which they felt physically or mentally unhealthy. An “unhealthy days” summary score was calculated, which included the number of days (ranging from 0 to 30) that an individual felt either mentally or physically unhealthy (or both).

#### Secondary Outcomes

##### Body Composition

The Tanita Body Composition Analyzer SC-331S was used to measure participants’ weight (to the nearest 0.1 kg) and body fat percentage via bioelectrical impedance analysis.

##### Dietary Intake

The 26-item NCI Dietary Screener Questionnaire (DSQ) [25] assessed diet quality. Participants responded to questions about the frequency and amount of selected food/drink intake over the past month, and estimates of intake values for fruits, vegetables, fiber, and added sugars were calculated.

##### Physical Activity

The 16-item Global Physical Activity Questionnaire (GPAQ) assessed the time participants spent in various physical activity intensities in the past week [26]. The total number of minutes per week spent sedentary and in moderate-to-vigorous leisure time physical activity was calculated.

##### Mindfulness

The 24-item Five Facet Mindfulness Questionnaire Short Form assesses five components of mindfulness: observing, describing, acting with awareness, nonjudging, and nonreactivity [27]. Participants rated the extent to which a variety of statements were true for them (eg, “I watch my feelings without getting carried away by them,” “I make judgments about whether my thoughts are good or bad”) on a 5-point Likert scale from 1 (never or very rarely true) to 5 (very often or always true). Items were summed, with higher scores indicating higher levels of mindfulness.

##### Stress

Participants reported their perceived stress via the 10-item Perceived Stress Scale (PSS-10), a survey that measures the extent to which life situations over the past month are viewed as uncontrollable, unpredictable, and stressful (eg, “In the past month, how often have you felt that things were going your way?”, “How often have you been upset because of something that happened unexpectedly?”) [28]. Participants rated items on a scale from 0 (never) to 4 (very often), and items were summed, with higher scores indicating higher levels of stress.

##### T2D Knowledge

An adapted version of the Diabetes Knowledge Test developed by Garcia et al [29] for Mexican American adults residing in South Texas was used to measure participants’ T2D knowledge. The adapted measure consisted of 26 items related to general T2D knowledge about diet, physical activity, and blood glucose monitoring (eg, “Shaking and sweating are signs of high blood sugar,” “The way I prepare my food is as important as the foods I eat,” “Regular exercise will increase the need for insulin or other diabetic medication”). Participants responded to items with “yes,” “no,” or “I don’t know,” and we calculated the percentage of items answered correctly.

##### T2D Management Self-Efficacy

The 15-item Diabetes Management Self-Efficacy Scale assessed participants’ confidence in their ability to engage in diabetes management activities (eg, “Avoid drinks that contain sugar,” “Exercise three times per week,” “Test blood glucose every day”) [30]. Items were rated on a scale from 1 (not at all confident) to 4 (completely confident), and an average score was calculated.

##### T2D Support Satisfaction

Participants were asked to rate how satisfied they were with T2D management support provided by four potential sources (ie, family support person, other family member, YMCA staff, and others) on a scale from 1 (very dissatisfied) to 6 (very satisfied) [31]. An average satisfaction score across all four categories was calculated.

### Participant Engagement

Four index variables (family engagement and support, participation in self-management education, program support and facilitation, and participation in self-monitoring) were created to track participants’ and family support persons’ engagement levels and uptake of key program activities. These index variables collectively represented the physical, social, and cognitive dimensions of influences on participants’ engagement in ¡Salud, Salud! [32]. *Family engagement and support* was determined by three activities: having a support person, completing at least one online lesson with a support person, and attending the orientation meeting with a support person. *Participation in self-management education* was determined by the number of core online health lessons (eight lessons) completed by the participants. *Program support and facilitation* was determined by the number of encounters participants had with the CHWs (11 coaching sessions) and the nutrition educator (1 meeting). *Participation in self-monitoring* was determined by the number of weekly surveys completed by the participants. The number of activities completed for each index variable was converted to percentages to standardize the dose and facilitate interpretation of the results.

### Statistical Analysis

Means (SDs) for continuous variables and frequencies and percentages for categorical variables were calculated for all participant characteristics and outcome measures. Paired *t*-tests were used to determine whether continuous primary and secondary outcome variables changed significantly from baseline to the 12-week assessment. McNemar chi-square tests were used to examine changes in the proportion of participants reporting good-to-excellent health from baseline to the 12-week assessment. Cohen d and φ estimated the effect sizes for continuous and categorical variables, respectively. The level of significance for all statistical tests was set at .05. Typically, adjustments to *P* values are applied to reduce type I errors in multiple comparisons. However, some statisticians argue that such adjustments may not be necessary in exploratory research, as they can decrease statistical power and increase the risk of type II errors, particularly in studies with small sample sizes [33]. In this study, sample sizes varied for each test due to the listwise deletion of missing values. Data were analyzed using IBM’s Statistical Package for the Social Sciences, version 28.0.

### Ethical Considerations

The study protocol was reviewed and approved by the Institutional Review Board at the University of Texas at Austin (approval number STUDY00002124). Written informed consent was obtained from all participants in either English or Spanish, depending on each participant’s preferred language. Participants were provided with a copy of the consent form, which included detailed information about the study, data collection, and their rights as participants. Data collection training for all study staff emphasized the importance of protecting participants’ privacy and maintaining confidentiality of data. Data collection, management, and quality assurance for this study were facilitated using tablets programmed with a Health Insurance Portability and Accountability Act (HIPAA)–compliant platform (ie, Connected Health) for participants and CHWs to track study activities, as well as REDCap, a secure data management system that ensures effective data collection, storage, retrieval, and quality control. All data collected in this study were deidentified, ensuring no identification of individual participants in the data or study was possible. As compensation for their participation, each participant received a 6-month family membership to the YMCA.

## Results

### Participant Characteristics

We distributed 2743 flyers and interacted with 3298 individuals through community outreach efforts. A total of 1798 individuals initiated the eligibility screener; however, only 363 (20.2%) completed it. Of those who completed the screener, 155 (42.7%) met the study’s eligibility criteria. We enrolled 81 (52.3%) participants with T2D or prediabetes (mean age 48.90 years, SD 12.57; n=57, 70.4%, female; n=66, 81.5%, Latino) who completed the baseline assessment and orientation session. The primary reasons why those eligible did not enroll were loss of interest or our inability to reach them after multiple attempts to complete baseline assessments. The analysis included only those participants with T2D or prediabetes who completed at least one of the primary and secondary outcome measures at the 12-week assessment (n=48, 59.3% retention).

Baseline participant characteristics are displayed in Table 2. Participants were predominantly Latino, female, and economically disadvantaged. Although 28 (58.3%) of 48 participants preferred to use the Spanish language in daily conversations, 33 (68.8%) were born outside the United States. In addition, 36 (75%) participants had physician-diagnosed T2D, and 34 (70.8%) had obesity (BMI≥30). At baseline, 30 (62.5%) participants had a designated support person when they enrolled, and 24 (88.9%) of 27 participants reported that they received diabetes management–related support in at least 1 of 23 areas from their designated support person, while 24 (60%) of 40 participants received support in at least 1 area from other family members.

We examined differences in participant characteristics and primary outcomes at baseline between those who completed and those who did not complete the 12-week assessment (results not shown). Those who completed the 12-week assessment were less likely to report having difficulty paying their bills (n=15, 31.3%) compared to noncompleters (n=18, 54.5%; *P*<.05). No other significant differences were found.

**Table 2:** Participant characteristics at baseline (N=48).

Characteristics		Participants, n (%)
**Race/ethnicity**		
	Latino	35 (72.9)
	White	6 (12.5)
	Black	2 (4.2)
	Asian	3 (6.3)
	Other	2 (4.2)
Female gender		35 (72.9)
Age≥50 years		23 (47.9)
Prefer the Spanish language		28 (58.3)
Currently not working		27 (56.3)
Difficulty paying bills		15 (31.3)
No health insurance		15 (31.3)
Less than high school education		30 (62.5)
Foreign born		33 (68.8)
Physician-diagnosed T2D^a^		36 (75.0)
Obesity^b^		34 (72.3)
Participated with a support person		30 (62.5)
Support in at least one area from support person^c^		24 (88.9)
Support in at least one area from other family member^d^		24 (60.0)

^a^T2D: type 2 diabetes.

^b^N=47.

^c^N=27.

^d^N=40.

### Participant Engagement in Program Activities

Table 3 shows the participant engagement in, and uptake of, key program activities across the four index variables. The highest level of engagement was in program support and facilitation, with participants completing 88% of ¡Salud, Salud! encounter sessions with CHWs and the nutrition counselor. Engagement levels in the other three indices were below 70%.

**Table 3:** Participant engagement in program activities.

Type of engagement	Mean (SD), range of completed activities	Mean proportion of activities completed (%)
Family engagement and support (number of support activities completed by the support person; maximum 3)	1.48 (1.22), 0-3	49
Participation in self-management training (number of online health lessons completed; maximum 8)	3.48 (3.14), 0-8	66
Program support and facilitation (number of encounters with study staff; maximum 13)	11.54 (2.10), 4-13	88
Participation in self-monitoring (number of weeks in which weekly self-monitoring surveys completed; maximum 12)	7.34 (3.75), 0-12	56

### Changes in Primary Outcomes

There was improvement in the primary outcomes from baseline to the12-week assessment. Although the reduction in HbA1c (–0.30%, *P*≤.09) was marginally significant (Table 4), the proportion of participants self-reporting good-to-excellent health significantly increased (two-sided *P*≤.003) from 19 (39.6%) at baseline to 28 (58.3%) at the 12-week assessment, with Somers D being 0.381 (SE 0.13), T being 2.93, and effect size (φ) being 0.39. Effect sizes were small for both outcomes.

**Table 4:** Comparisons of changes in the study’s primary and secondary outcomes from baseline to 12-week assessment.

Outcomes	Value, mean (SD)			Change (SE; 95% CI) from baseline to 12-week assessment		Paired *t*-test results			Effect size (Cohen d)
	Baseline	12 weeks			*t* (*df*)		*P* value		
HbA1c^a^ (%)^b^	7.33 (1.78)	7.04 (1.58)	–0.30 (0.17; –0.64 to 0.05)		–1.71(40)		.09	–0.27	
Body weight (kg)^b^	89.51 (20.17)	88.21 (19.74)	–1.30 (0.55; –2.41 to –0.18)		–2.36(40)		.02	–0.37	
Body fat (%)^b^	41.20 (6.47)	39.93 (6.58)	–1.26 (0.55; –2.37 to –0.16)		–2.31(40)		.01	–0.36	
Diabetes knowledge^c^	16.94 (4.39)	17.89 (3.60)	0.94 (0.56; –0.19 to 2.08)		1.69(35)		.10	0.29	
Diabetes management self-efficacy^d^	2.84 (0.64)	3.05 (0.58)	0.21 (0.12; –0.03 to 0.45)		1.77(38)		.08	0.28	
Mindfulness^e^	30.66(5.89)	32.87(6.05)	2.21 (0.88; 0.44 to 3.98)		2.51(46)		.01	0.37	
Perceived stress^e^	1.28 (0.76)	1.00 (0.79)	0.28 (0.12; –0.51 to –0.04)		–2.35(46)		.02	–0.34	
Unhealthy days^f^	7.26 (9.46)	4.87 (5.80)	2.39(1.73; –1.11 to 5.90)		1.39(37)		.17	–0.23	
Satisfaction with support^g^	4.70 (1.62)	4.99 (1.32)	–0.30 (0.34; –0.99 to 0.40)		–0.88(34)		.39	0.15	
Sedentary time (minutes/week)^b^	287.44 (248.00)	217.17 (213.00)	–70.27 (23.87; –118.52 to –22.02)		–2.94 (40)		.003	–0.46	
Leisure time moderate-to-vigorous activity (minutes/week)^h^	55.86 (95.79)	60.86 (91.63)	5.00 (22.64; –41.38 to 51.38)		0.22(28)		.82	0.04	
Fiber intake (g/day)^i^	15.97 (2.61)	15.29 (2.44)	–0.68 (0.37; –1.41 to 0.06)		–1.85 (45)		.07	–0.27	
Added sugar intake (teaspoons/day)^j^	13.76 (3.34)	11.61 (1.06)	–2.15 (0.46; –3.08 to –1.23)		–4.70(42)		<.001	–0.71	
Vegetable intake (cups/day)^i^	1.32 (0.37)	1.25 (0.25)	–0.07 (0.06; –0.18 to 0.04)		–1.28(45)		.21	–0.19	

^a^HbA1c: hemoglobin A1c.

^b^N=41.

^c^N=36.

^d^N=39.

^e^N=47.

^f^N=38.

^g^N=35.

### Changes in Secondary Outcomes

Table 4 also shows changes in secondary outcomes from baseline to 12 weeks. Significant reductions in body weight (*P*≤.02) and body fat percentage (*P*≤.01) were observed. Increases in Diabetes Knowledge Test scores (*P*=.10) and Diabetes Self-Management Self-Efficacy Scale scores (*P*=.08) were marginally significant. PSS-10 scores significantly reduced (*P*=.02), and mindfulness scores significantly improved (*P*=.001). Although there were improvements in the number of physically and mentally unhealthy days during the past month (–2.39, SE 10.65) and in satisfaction scores for support across all support person categories (0.30, SE 2.00), these improvements were not statistically significant. Additionally, although the time spent in sedentary activities significantly reduced (–70 minutes/day, *P*=.003), no change in leisure time physical activity was observed. Finally, the added sugar intake significantly reduced (*P*≤.001), and fiber intake marginally increased (*P*≤.07). No change was reported in vegetable intake. Effect sizes were small for all secondary outcomes, except for a medium effect size for added sugar intake.

## Discussion

### Principal Findings

The multilevel and multidomain ¡Salud, Salud! program demonstrated promising but modest effects in supporting T2D self-management practices among predominantly low-income Latino adults with T2D or prediabetes. Our findings partially supported the study hypothesis, showing a marginally significant reduction in HbA1c (–0.30%, *P*≤.09) and significant improvement in self-reported general health (*P*≤.003) over the 12-week program. Importantly, participants demonstrated significant improvements in secondary outcomes, including body weight, psychological well-being, sedentary behavior, and added sugar intake, although the effect sizes were generally small.

To increase the uptake of T2D self-management practices, ¡Salud, Salud! was designed following national standards for DSMES that incorporated evidence-based strategies for individuals experiencing health disparities [34]. These strategies included focusing on lifestyle behavior changes, removing barriers to physical activity, using telemedicine, providing support and facilitation by lay community educators, setting patient-centered goals, and implementing stress management [22]. Additionally, digital technologies were used to increase the program’s accessibility and availability with timely and personalized coaching by trained CHWs [35]. Nonetheless, modest effects in glycemic control and weight loss among ¡Salud, Salud! participants are consistent with the literature on translation studies of evidence-based T2D management and prevention programs, such as Look AHEAD and the Diabetes Prevention Program [36,37]. The current literature demonstrates that participation in DSMES among people with T2D or prediabetes results in small-to-moderate reductions in HbA1c and body weight in in-person programs [38,39], while digitally delivered programs tend to produce small effects [13,40]. Furthermore, reductions in HbA1c and weight are smaller in studies with low-income minority participants compared to White participants [39,41]. For example, a 2020 systematic review reported a reduction of –0.24% in HbA1c among Latino participants in DSMES programs [42]. Additionally, moderate levels of program engagement in three of the four engagement index variables might have contributed to the smaller reduction in HbA1c among ¡Salud, Salud! participants. Previous research demonstrates that Latinos in low-to-medium-intensity DSMES interventions had similar levels of reduction in HbA1c [13]. Despite ongoing efforts at cultural tailoring and adaptation [43], the inequity in program effectiveness among socioeconomically disadvantaged minority populations is alarming [44] and requires innovations that address the social determinants of diabetes care to address upstream factors, such as living in deprived neighborhoods, competing priorities and time constraints, and access to affordable healthy foods—all of which influence T2D self-management at the individual level [45,46]. A 2024 systematic review of 106 randomized controlled trials of DSMES interventions among socially marginalized racial and ethnic minority populations in the United States noted that relatively few interventions target policy, community resources, or the physical environment, but those that do show more promise than DSMES alone [15].

Our findings regarding participants’ quality of life and psychological well-being are noteworthy. The significant improvement in self-reported general health and psychological measures addresses a critical need, as individuals with T2D typically report a lower quality of life due to the burden associated with disease management [47]. These improvements may be especially meaningful for our socioeconomically disadvantaged participants, who often face additional stressors that can complicate T2D management [48,49].

Although participation in DSMES has contributed to improvements in T2D self-management knowledge, self-efficacy, and practices [17,38], the integration of family support in DSMES can have a greater impact on T2D care, with positive effects on diabetes-related support, self-efficacy, psychological well-being, and behaviors (eg, physical activity, diet) [18]. Although ¡Salud, Salud! was designed to be family centered, the program’s effect on diabetes care–related social and psychological outcomes was mixed. We observed marginally significant improvements in DSM knowledge and self-efficacy, significant improvements in fiber intake and reduced time spent sedentary, but no improvement in physical activity or vegetable intake. Additionally, ¡Salud, Salud! participants reported marginally significant improvements in satisfaction with support in T2D care. Notably, for participants who reported receiving T2D care support from their support person or another family member at baseline, satisfaction levels were already high before enrolling in ¡Salud, Salud!, potentially contributing to the marginal effect of the intervention on satisfaction with support [50]. However, the level of family engagement and support in ¡Salud, Salud! (ie, activities completed by the support person) was less than 50%, suggesting a lack of instrumental family support related to T2D management. Therefore, it is critical to investigate how to enhance family informational and instrumental support in socioeconomically disadvantaged populations with T2D [9,18].

Participants were most engaged with the CHW encounters, indicating that our participants relied on human interaction more than on the self-guided mHealth components. In future studies, it might be advisable to increase digital literacy and navigation support through additional staff and CHW training and dedicated time when working with this population [32]. Although our CHWs were available to assist with the technology, their time with each participant was limited and more focused on goal setting and lifestyle modification support.

### Strengths and Limitations

We attribute the promising impacts of ¡Salud, Salud! to the adoption of evidence-based strategies for DSMES [22,51], including the National Standards for Culturally and Linguistically Appropriate Services in Health and Health Care [52], to meet the needs of our study participants. Other strengths of the study included a theory-driven intervention model, community partnership with the YMCA that provided venues to reach the community, CHW facilitation and support that had the highest level of participant engagement, and program delivery combining digital technologies and personalized live coaching that are desired by socioeconomically disadvantaged populations [53].

Several important limitations should be considered in the interpretation of the study results. First, the short duration of the study (ie, 12 weeks) likely limited ¡Salud, Salud!’s impact on HbA1c and body weight, as DSMES interventions typically last 6-12 months and are often supplemented with maintenance programs [54,55]. Second, although the study sample was powered to test the study hypotheses, low enrollment at baseline and high participant dropout led to a smaller sample at posttest. Low participation [12] and high dropout rates [36,55] in clinical trials testing DSMES programs have been reported among socioeconomically disadvantaged participants. However, this limitation raises concerns about the reliability and generalizability of the findings, even though there were no differences between the completers and noncompleters in the study. Third, we were not able to evaluate the effect of family engagement on the study outcomes, given the low level of family participation and small sample size. Finally, ¡Salud, Salud! was not designed to address the social and financial needs that can influence the uptake of T2D care–related practices, such as access to affordable food resources and lack of transportation to exercise at the YMCA [32,56].

### Conclusion

The ¡Salud, Salud! program demonstrated promising but modest effects in improving T2D-related outcomes among predominantly low-income Latinos with T2D. Findings from this study reveal that cultural tailoring of DSMES is necessary but insufficient to optimize the uptake of evidence-based strategies and enhance participant engagement in T2D self-management practices. Future studies should broaden the search for effective strategies to reduce the barriers influencing the uptake of evidence-based DSM practices in low-resource populations.

## Abbreviations

CHW: community health worker
DSM: diabetes self-management
DSMES: diabetes self-management education and support
HbA1c: hemoglobin A1c
HRQoL: health-related quality of life
IMB: information-motivation-behavioral skills
mHealth: mobile health
PSS-10: 10-item Perceived Stress Scale
T2D: type 2 diabetes
YMCA: Young Men’s Christian Association

## Data Availability

The datasets used and analyzed during the study are available from the corresponding author upon request.
